# GABA_A_ and NMDA receptor density alterations and their behavioral correlates in the gestational methylazoxymethanol acetate model for schizophrenia

**DOI:** 10.1038/s41386-021-01213-0

**Published:** 2021-11-06

**Authors:** Amanda Kiemes, Felipe V. Gomes, Diana Cash, Daniela L. Uliana, Camilla Simmons, Nisha Singh, Anthony C. Vernon, Federico Turkheimer, Cathy Davies, James M. Stone, Anthony A. Grace, Gemma Modinos

**Affiliations:** 1 Department of Psychosis Studies, Institute of Psychiatry, Psychology and Neuroscience, King’s College London, London, UK; 2 Department of Pharmacology, Ribeirão Preto Medical School, University of São Paulo, Ribeirão Preto, Brazil; 3 Department of Neuroimaging, Institute of Psychiatry, Psychology and Neuroscience, King’s College London, London, UK; 4 Departments of Neuroscience, Psychiatry and Psychology, University of Pittsburgh, Pittsburgh, PA USA; 5 Department of Psychiatry, University of Oxford, Warneford Hospital, Oxford, UK; 6 Department of Basic and Clinical Neuroscience, Institute of Psychiatry, Psychology and Neuroscience, King’s College London, London, UK; 7 MRC Centre for Neurodevelopmental Disorders, King’s College London, London, UK; 8 Brighton and Sussex Medical School, University of Sussex, Brighton, UK

**Keywords:** Psychosis, Schizophrenia, Experimental models of disease, Schizophrenia, Psychosis

## Abstract

Hippocampal hyperactivity driven by GABAergic interneuron deficits and NMDA receptor hypofunction is associated with the hyperdopaminergic state often observed in schizophrenia. Furthermore, previous research in the methylazoxymethanol acetate (MAM) rat model has demonstrated that repeated peripubertal diazepam administration can prevent the emergence of adult hippocampal hyperactivity, dopamine-system hyperactivity, and associated psychosis-relevant behaviors. Here, we sought to characterize hippocampal GABA_A_ and NMDA receptors in MAM-treated rats and to elucidate the receptor mechanisms underlying the promising effects of peripubertal diazepam exposure. Quantitative receptor autoradiography was used to measure receptor density in the dorsal hippocampus CA1, ventral hippocampus CA1, and ventral subiculum. Specifically, [^3^H]-Ro15-4513 was used to quantify the density of α5GABA_A_ receptors (α5GABA_A_R), [^3^H]-flumazenil to quantify α1-3;5GABA_A_R, and [^3^H]-MK801 to quantify NMDA receptors. MAM rats exhibited anxiety and schizophrenia-relevant behaviors as measured by elevated plus maze and amphetamine-induced hyperlocomotion (AIH), although diazepam only partially rescued these behaviors. α5GABA_A_R density was reduced in MAM-treated rats in all hippocampal sub-regions, and negatively correlated with AIH. Ventral hippocampus CA1 α5GABA_A_R density was positively correlated with anxiety-like behavior. Dorsal hippocampus CA1 NMDA receptor density was increased in MAM-treated rats, and positively correlated with AIH. [^3^H]-flumazenil revealed no significant effects. Finally, we found no significant effect of diazepam treatment on receptor densities, potentially related to the only partial rescue of schizophrenia-relevant phenotypes. Overall, our findings provide first evidence of α5GABA_A_R and NMDA receptor abnormalities in the MAM model, suggesting that more selective pharmacological agents may become a novel therapeutic mechanism in schizophrenia.

## Introduction

Hippocampal dysfunction has been proposed to underlie the subcortical hyperdopaminergic state commonly associated with psychotic disorders such as schizophrenia [1, 2]. Human postmortem studies revealed reduced hippocampal volume and morphological changes in patients with schizophrenia compared to controls [3, 4]. Consistent with these observations, in vivo neuroimaging studies have also documented hippocampal volume reductions [5, 6], and functional abnormalities involving increased hippocampal metabolism, blood flow, and activation [7–9]. Moreover, alterations in hippocampal morphology and function have also been observed in individuals at high risk for psychosis [10–13], suggesting that hippocampal dysfunction is already present in psychosis vulnerability states.

Prevailing theories suggest hippocampal hyperactivity in schizophrenia is due to deficits in GABAergic inhibition [1, 2, 14]. In particular, GABAergic abnormalities in the hippocampus have been found in patient postmortem samples, including lower levels of the GABA synthesizing enzyme GAD67 [15], and a functional loss of parvalbumin-expressing interneurons [16]. This functional loss of parvalbumin-expressing interneurons is thought to result in decreased inhibitory regulation of glutamatergic (excitatory) pyramidal neuron activity, as they synapse onto cell bodies and axon initial segments of pyramidal neurons [17, 18]. Reduced synthesis and release of GABA has been proposed to also lead to a compensatory increase of GABA receptors [19]. Endogenous GABA can act on two different types of receptors, GABA_A_ (GABA_A_R), and GABA_B_ receptors. GABA_A_R are characterized by a wide range of structural diversity and region-specific distribution, due to the large variety of GABA_A_R subunit types [20]. For example, classical benzodiazepines have a high affinity for GABA_A_R at the benzodiazepine binding site (GABA_A_-BZR) at the junction between α1, α2, α3, or α5 subunits and the γ2 subunit, and a low affinity for most other receptor subtypes [21, 22]. Within the hippocampus, α5 subunit-containing GABA_A_Rs (α5GABA_A_R) make up 25% of GABA_A_R in this region [23], but <5% of all GABA_A_R in the brain [20]. Additionally, this receptor is critically involved in stress responsivity and sensorimotor gating [20], both commonly disrupted in schizophrenia and high-risk states [24–26]. In postmortem tissue samples of patients with schizophrenia, GABA_A_R were found to be increased in the hippocampus, as measured with quantitative receptor autoradiography using [^3^H]-muscimol [27]. Postmortem studies, however, may be influenced by effects of long-term exposure to medication [28, 29] and illness chronicity on brain tissue as well as technical challenges of tissue condition and preservation. In this context, however, results from in vivo neuroimaging studies using positron emission tomography (PET) and ligands that bind to GABA_A_-BZR ([^123^I]-iomazenil, [^18^F]-fluoroflumazenil, and [^11^C]-flumazenil) have been inconsistent [30]. Interestingly, the inverse agonist [^11^C]-Ro15-4513 enables measurement of α5GABA_A_R availability more selectively and has also been used in schizophrenia research. From the two existing [^11^C]-Ro15-4513 studies, one including medicated and non-medicated schizophrenia patients found no significant effects [31], while a more recent study reported lower α5GABA_A_R availability in unmedicated patients only [28].

Preclinical research has provided additional evidence for the relationship between GABAergic abnormalities, hippocampal hyperactivity and the hyperdopaminergic state. A well-validated rat model of relevance for schizophrenia, the methylazoxymethanol acetate (MAM) gestational day (GD) 17 [32] model, introduces a neurodevelopmental insult in the offspring of MAM-treated dams. Their offspring display several behavioral, neuroanatomical, and electrophysiological deficits which recapitulate hallmark schizophrenia-relevant features [33]. These, for example, include amphetamine-induced hyperlocomotion (AIH) indexing increased striatal dopamine activity [34–36], and anxiety-like behavior in the elevated plus maze (EPM) [35, 37, 38]. MAM model studies also suggest that hippocampal hyperactivity results from a functional loss of parvalbumin-expressing interneurons [39]. Tonic inhibition provided through α5GABA_A_R provides crucial regulation of glutamatergic pyramidal neuron activity [40]. Importantly, systemic administration of an α5GABA_A_R positive allosteric modulator to MAM-treated adult rats normalized dopamine signaling and reduced the AIH abnormalities [34], and overexpression of hippocampal α5GABA_A_R via viral-mediated gene transfer was shown to also improve schizophrenia-relevant behaviors in the MAM model [41]. With important implications for prophylactic psychiatry, repeated administration of the anxiolytic drug diazepam to MAM rats during the peripubertal period prevented the emergence of parvalbumin-expressing interneuron loss [38], subcortical hyperdopaminergia and elevated stress response [35] in adulthood. Diazepam, a classical benzodiazepine binding to GABA_A_-BZR [22], enhances GABAergic signaling by increasing the affinity of GABA and increasing GABA_A_R channel opening frequency [42], but its wider activity profile may involve acting on both tonic and phasic inhibition within the hippocampus [43]. However, the molecular mechanisms by which diazepam enacts its preventative effects on schizophrenia-relevant pathology in the MAM model is unknown.

The present study aimed to address this issue by using quantitative receptor autoradiography to characterize hippocampal GABAergic and glutamatergic receptor systems in the context of MAM pathophysiology, and the potential modulatory effects of repeated peripubertal diazepam administration on these systems. Specifically, we focused on α5GABA_A_R, GABA_A_-BZR (α1-3;α5), and NMDA receptor (NMDAR) density. To increase translational relevance, associations between receptor density and behavioral correlates relevant to schizophrenia were examined (i.e., anxiety in the EPM and AIH). Given prior animal research implicating the α5 subunit in the pathophysiology and rescue of schizophrenia-relevant deficits [34, 41, 44, 45], we hypothesized that MAM-treated rats would show reduced α5GABA_A_R binding in the hippocampus, particularly in the ventral portion [46, 47], and that this deficit would be rescued by diazepam treatment. We further hypothesized that unspecific GABA_A_-BZR would be unaltered in MAM-treated rats but increased by diazepam treatment. Finally, we hypothesized NMDAR density would be increased in MAM-treated rats based on prior human postmortem studies [48], but that it would remain unaffected by diazepam treatment. Because of prior research implicating the CA1 subfield of the ventral hippocampus (vHipp CA1) [19, 49] and the ventral subiculum of the hippocampus (vSub) [46] in the pathogenesis of psychosis, we focused on these regions. Additionally, we explored the CA1 subfield of the dorsal hippocampus (dHipp CA1), given previous evidence demonstrating alterations of GABA_A_R in this region through antipsychotic exposure [29].

## Methods and materials

### Animals

All experiments were conducted in accordance with the USPHS’s Guide for the Care and Use of Laboratory Animals and were approved by the Institutional Animal Care and Use Committee of the University of Pittsburgh. Pregnant Sprague-Dawley dams were obtained on GD15 (Envigo, Indianapolis, IN) and injected with saline (SAL) or MAM (20 mg/kg, intraperitoneal (i.p.); Midwest Research Institute, Kansas City, MO) on GD17. Animals were housed in a 12 h light/dark cycle (lights on at 7am) in a temperature- (22 ± 1 °C) and humidity-controlled environment.

### Experimental design

Male pups were weaned on postnatal day (PD) 21 and housed two to three per cage. Animals received diazepam (DZP) or vehicle (VEH) once daily during the peripubertal period (PD31-40). Litters from 7 MAM- and SAL-treated dams each were divided and assigned to the diazepam (SAL:DZP, *n* = 20; MAM:DZP, *n* = 17) and vehicle (SAL:VEH, *n* = 19; MAM:VEH, *n* = 15) group. Once animals reached adulthood (PD62), they underwent behavioral experiments: (i) EPM to examine anxiety responses, followed by (ii) AIH to test sensitivity to psychostimulants as a proxy for whether MAM exposure had been effective. Subsequently, brains were collected for autoradiography. All animals underwent behavioral testing and autoradiography.

### Oral administration of diazepam

Diazepam was administered orally based on a previous study [35]. Briefly, diazepam (5 mg/kg, Hospira, INC., Lake Forest, IL) was delivered in wafers (mini Nilla Wafers; Kraft Food) and topped with liquid sugar and sweetened condensed milk (Eagle Brand).

### Elevated plus maze (EPM)

The EPM (San Diego Instruments, San Diego, CA) consisted of four 50 cm long and 10 cm wide elevated arms in a cross-like shape positioned 50 cm above the floor. Two opposite arms were enclosed by 40 cm high opaque walls, and the other two were without any walls. A central platform (10 × 10 cm^2^) connected the four arms. Rats were habituated to the testing room for 1 h before the test. For the test, each rat was placed on the central platform, facing an enclosed arm. Behavior was recorded for 5 min. The arena was cleaned between rats with ethanol 70% v/v. Both the percentage of time spent in the open arms and the percentage of entries into the open arms were taken as measurements of anxiety-like behavior.

### Amphetamine-induced hyperlocomotion (AIH)

Rats were placed into an open-field arena (Coulbourn Instruments, Allentown, PA). Animals’ spontaneous locomotor activity was recorded for 30 min before being injected with D-amphetamine sulfate (0.5 mg/kg, i.p.) after which their locomotor activity was recorded for another 90 min. Locomotor activity was measured via beam breaks and recorded with TruScan software (Coulbourn Instruments). The arena was cleaned between rats with ethanol 70% v/v.

### Tissue preparation

Rats were anaesthetized with isoflurane (Covetrus, Dublin, OH) and decapitated at PD69. Brains were carefully removed, and flash frozen in isopentane at −40 °C. Brains were shipped frozen on dry ice to the BRAIN Center (Institute of Psychiatry, Psychology & Neuroscience, London, UK), where coronal sections (20 µm thick) were cut in series using a cryostat (Leica CM1950). Sections were mounted onto gelatin coated glass slides and stored at −80 °C until used for autoradiography.

### Quantitative receptor autoradiography

Quantitative autoradiography with the radiotracers [^3^H]-Ro15-4513 and [^3^H]-flumazenil was conducted as described previously [29]. To quantify α5GABA_A_R density, we used [^3^H]-Ro15-4513 (Perkin Elmer, NET925250UC). This radiolabeled ligand has a 10- to 15-fold higher affinity to α5GABA_A_R compared to remaining subtypes [50], yielding a high selectivity (60–70%) for the α5 subunit [51] with a smaller portion of selectivity for the α1 subunit [52]. This ligand’s binding pattern highly covaries with the expression of GABRA5, the gene encoding for the α5 subunit [53]. To quantify nonspecific binding, we used bretazenil (Sigma, B6434-25MG). Sections were preincubated in Tris buffer (50 mM) for 20 min. Slides were subsequently incubated in either 2 nM [^3^H]-Ro15-4513 in Tris buffer for specific binding, or 10 µM bretazenil with 2 nM [^3^H]-Ro15-4513 in Tris buffer for nonspecific binding for 60 min. Sections were washed with Tris buffer (2 × 2 min), dipped in distilled water and laid out to dry overnight. All solutions were at room temperature. Dry slides were placed into light-proof cassettes alongside a radioactive [^3^H]-standards slide (American Radiolabelled Chemicals, Inc., USA, ART-123A). A [^3^H]-sensitive film (Amersham Hyperfilm, 28906845) was placed on top of the radioactive slides and exposed for 8 weeks. The film was subsequently developed with a Protex Ecomax film developer (Protec GmbH & Co, Germany).

Similar procedures were used with [^3^H]-flumazenil (Perkin Elmer, NET757001MC), which was used to quantify GABA_A_-BZR binding [54]. Solutions concentrations were 1 nM [^3^H]-flumazenil for total binding, and 10 µM flunitrazepam (Sigma Aldrich, F-907 1 ML) with 1 nM [^3^H]-flumazenil for nonspecific binding. These were incubated for 60 min and washed in Tris buffer both at 4 °C. Film exposure time was 4 weeks long.

To quantify NMDAR, [^3^H]-MK801 (Sigma, M107-25MG) was used with similar procedures as above. Slides were incubated in a 5 nM [^3^H]-MK801 solution for total binding, or in a 5 nM [^3^H]-MK801 solution with the addition of 10 µM MK801 for nonspecific binding. Incubation time was 120 min at room temperature. Dried slides were exposed to film for 4 weeks.

### Quantification of receptor binding

**Fig. 1:**
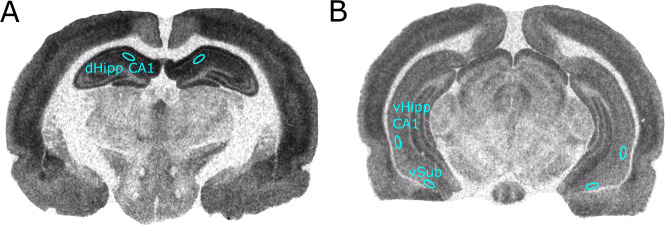
Representative [^3^H]-Ro15-4513 autoradiograph displaying ROI placement. The same ROI placement was used for [^3^H]-flumazenil and [^3^H]-MK801. **A** dorsal hippocampus CA1 (dHipp CA1). **B** ventral hippocampus CA1 (vHipp CA1), ventral subiculum (vSub).

Developed films were captured using a Nikon SLR camera. Images of [^3^H]-Ro15-4513 and [^3^H]-flumazenil binding were preprocessed (see Supplementary Methods). Using MCID software (Imaging Research Inc., 2003), we sampled optical density (OD) values from three primary regions of interests (ROIs) bilaterally (Fig. 1): dHipp CA1, vHipp CA1, and vSub. Four secondary ROIs were also sampled (Fig. S1). Anatomical regions were defined with the use of Paxinos and Watson’s rat brain atlas [55]. Receptor binding (nCi/mg) was calculated with robust regression interpolation in GraphPad Prism (v9.1.1 for Macintosh, Graphpad Software, La Jolla, CA) using standard curves created from OD measurements of [^3^H]-standards slide for each film. Nonspecific binding for [^3^H]-Ro15-4513 and [^3^H]-flumazenil were negligible (Fig. S2). [^3^H]-MK801 nonspecific binding was subtracted from total binding values to calculate [^3^H]-MK801 specific binding.

### Statistical analysis

Statistical analysis was performed using GraphPad Prism software (v9.1.1 for Macintosh). Due to failures of the TruScan software, one animal was excluded from analysis for the AIH. Further animals were excluded in the autoradiography data due to failures of the autoradiography protocol: nine animals were excluded for [^3^H]-Ro15-4513 and [^3^H]-flumazenil and two animals were excluded for [^3^H]-MK801 (see Supplementary Table S1 for *n*-values per group). To analyze main effects of group (MAM/SAL) and condition (DZP/VEH), two-way ANOVAs (AIH total movement; EPM) were utilized. For the AIH time-course and the autoradiography data, three-way mixed ANOVAs were used with group (MAM/SAL) and condition (DZP/VEH) as between-group factors and time (for AIH) or ROI (for autoradiography data) as within-group factor. ROI interaction effects with group were followed up with two-way mixed ANOVA (between-subject factor: group; repeated measure: ROI). Pearson’s correlations between behavioral measures and receptor density measures were run where autoradiography data showed significant effects. For these analyses, all rats from different groups were pooled together (see correlational values per group in Supplementary Table 2). Post hoc tests were performed where appropriate, corrected using Benjamini–Hochberg method (significance set at *q* < 0.05*)* [56]. The significance threshold was set to *p* < 0.05 for all other analyses.

## Results

### MAM-induced behavioral phenotypes were partially rescued by peripubertal diazepam

**Fig. 2:**
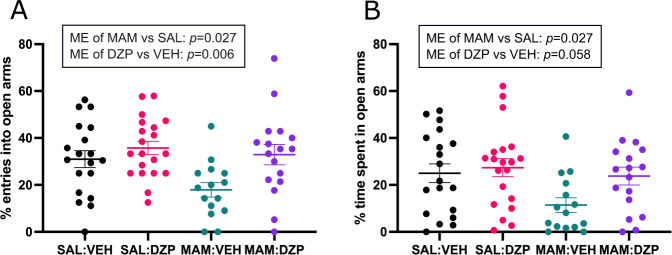
Anxiety-like behavior in the EPM (*n* = 15–20/group). **A** Percentage of entries into open arms. A two-way ANOVA showed a main effect of group (MAM vs. SAL; *F*_(1,67)_ = 5.146, *p* = 0.027) and of condition (DZP vs. VEH; *F*_(1,67)_ = 7.914, *p* = 0.006). **B** Percentage of time spent in the open arms. A two-way ANOVA showed a significant main effect of group (MAM vs. SAL; *F*_(1,67)_ = 5.088, *p* = 0.027), but a main effect of condition failed to reach significance (DZP vs. VEH; *F*_(1,67)_ = 3.729, *p* = 0.058). Data are displayed as mean ± SEM.

Adult MAM rats were previously shown to have a heightened anxiety response in the EPM [35, 37, 38]. This anxiety-like phenotype was once again confirmed by both measures: MAM-treated rats showed less entries into open arms (main effect of group: *F*_(1,67)_ = 5.146, *p* = 0.027, *η*^2^ = 0.071; Fig. 2A), and less time spent in open arms (main effect of group: *F*_(1,67)_ = 5.088, *p* = 0.027, *η*^2^ = 0.071; Fig. 2B). Anxiety-like behavior was significantly decreased by peripubertal diazepam treatment in the entries into open arms measure (main effect of condition: *F*_(1,67)_ = 7.914, *p* = 0.006, *η*^2^ = 0.106; Fig. 2A), but failed to reach statistical significance for the time spent in open arms measure (main effect of condition: *F*_(1,67)_ = 3.729, *p* = 0.058, *η*^2^ = 0.053; Fig. 2B). No interaction effects were observed for either measure (*p* > 0.05).

**Fig. 3:**
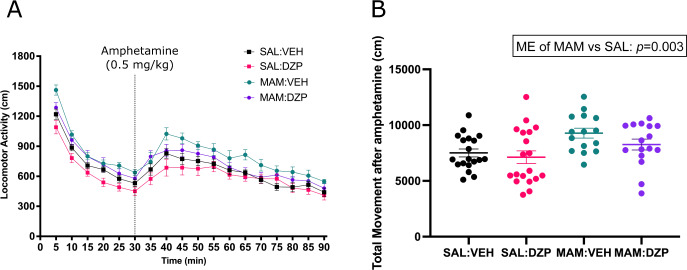
MAM rats showed increased locomotor response to amphetamine compared to controls, which was not prevented by peripubertal diazepam treatment (5 mg/kg, oral; daily PD31-40; *n* = 15–20/group). **A** Time-course of locomotor activity over 90 min. **B** Total movement postamphetamine injection. A two-way ANOVA showed a main effect of group (MAM vs. SAL; *F*_(1,65)_ = 9.282, *p* = 0.003) but no main effect of condition (DZP vs. VEH; *F*_(1,65)_ = 2.163, *p* = 0.146). Data are displayed as mean ± SEM.

Consistent with previous studies [34–36], MAM-treated adult offspring exhibited increased locomotion in response to amphetamine as compared to SAL-treated adult rats (main effect of group: *F*_(1,65)_ = 9.631, *p* = 0.003, *η*^2^ = 0.156; Fig. 3A). No main effect of condition (*F*_(1,65)_ = 2.609, *p* = 0.111, *η*^2^ = 0.051) or interaction effects (*p* > 0.05) were found. Total movement distance postamphetamine injection further corroborated a significant main effect of group, with increased locomotion in MAM rats (*F*_(1,65)_ = 9.282, *p* = 0.003, *η*^2^ = 0.125; Fig. 3B), but similarly found no effect of condition (*F*_(1,65)_ = 2.163, *p* = 0.146; *η*^2^ = 0.032) or interaction effect (*F*_(1,65)_ = 0.454, *p* = 0.503, *η*^2^ = 0.007).

### MAM treatment produced aberrant α5GABA_A_ and NMDA receptor density in adult rats

**Fig. 4:**
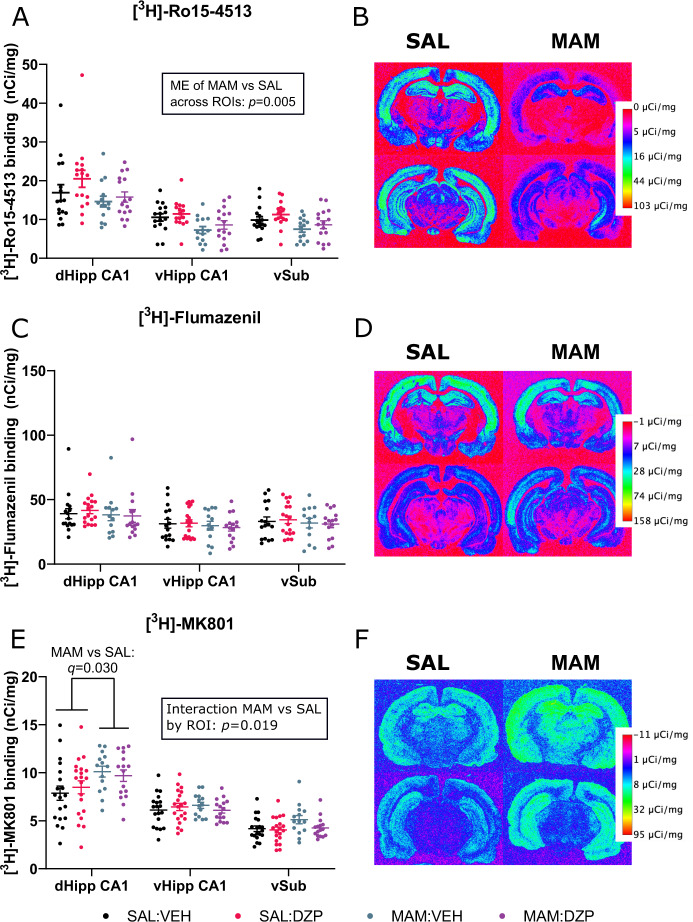
Autoradiography results. **A** A mixed three-way ANOVA of [^3^H]-Ro15-4513 binding (*n* = 14–16/group) showed a main effect of group (MAM vs. SAL; *F*_(1,58)_ = 8.410, *p* = 0.005) but no main effect of condition (DZP vs. SAL; *F*_(1,58)_ = 2.303, *p* = 0.135). **C** A mixed three-way ANOVA of [^3^H]-flumazenil binding (*n* = 13–18/group) showed no main effects (*p* > 0.05). **E** A mixed three-way ANOVA of [^3^H]-MK801 binding (*n* = 15–20/group) showed a ROI × group interaction (*F*_(2,130)_ = 4.117, *p* = 0.019) and follow-up analysis revealed a MAM vs. SAL difference in the dHipp CA1 (*p* = 0.001, *q* = 0.030). Pseudo-color representative autoradiographs of (**B**) [^3^H]-Ro15-451^3^ binding, (**D**) [^3^H]-flumazenil binding, and (**F**) [^3^H]-MK801 binding. Data are displayed as mean ± SEM.

α5GABA_A_R density, as indexed by [^3^H]-Ro15-4513 binding, showed a significant main effect of group, with lower binding in MAM rats compared to SAL (*F*_(1, 58)_ = 8.410, *p* = 0.005, *η*^2^ = 0.127; Fig. 4A). There was no significant effect of condition (*F*_(1,58)_ = 2.303, *p* = 0.135, *η*^2^ = 0.038) and no interaction effects were identified (*p* > 0.05).

General GABA_A_-BZR density, as measured with [^3^H]-flumazenil binding, showed no difference between MAM and SAL groups (*F*_(1,58)_ = 0.667, *p* = 0.417, *η*^2^ = 0.011), no effect of condition (DZP vs. VEHe) (*F*_(1,58)_ = 0.004, *p* = 0.952, *η*^2^ < 0.001; Fig. 4B) and no interaction effects (*p* > 0.05).

Finally, in terms of [^3^H]-MK801 binding, a main effect of group was observed, with greater binding in MAM compared to SAL (*F*_(1,65)_ = 5.483, *p* = 0.022, *η*^2^ = 0.078; Fig. 4C), but no overall effect of condition (*F*_(1,65)_ = 0.233, *p* = 0.631, *η*^2^ = 0.004). Furthermore, an ROI × group interaction effect was found (*F*_(2,130)_ = 4.117, *p* = 0.019, *η*^2^ = 0.060). Follow-up two-way mixed ANOVA, where the SAL:VEH and SAL:DZP groups were combined into one SAL group and the MAM:VEH and MAM:DZP groups into one MAM group, reflected that the effect of group was only significant within the dHipp CA1 region (greater [^3^H]-MK801 binding in MAM vs. SAL, *p* = 0.001, *q* = 0.030). The above autoradiographic analyses were repeated with secondary ROIs included (see Supplementary Results).

### α5GABA_A_R and NMDAR density were differentially correlated with schizophrenia-relevant behaviors

**Fig. 5:**
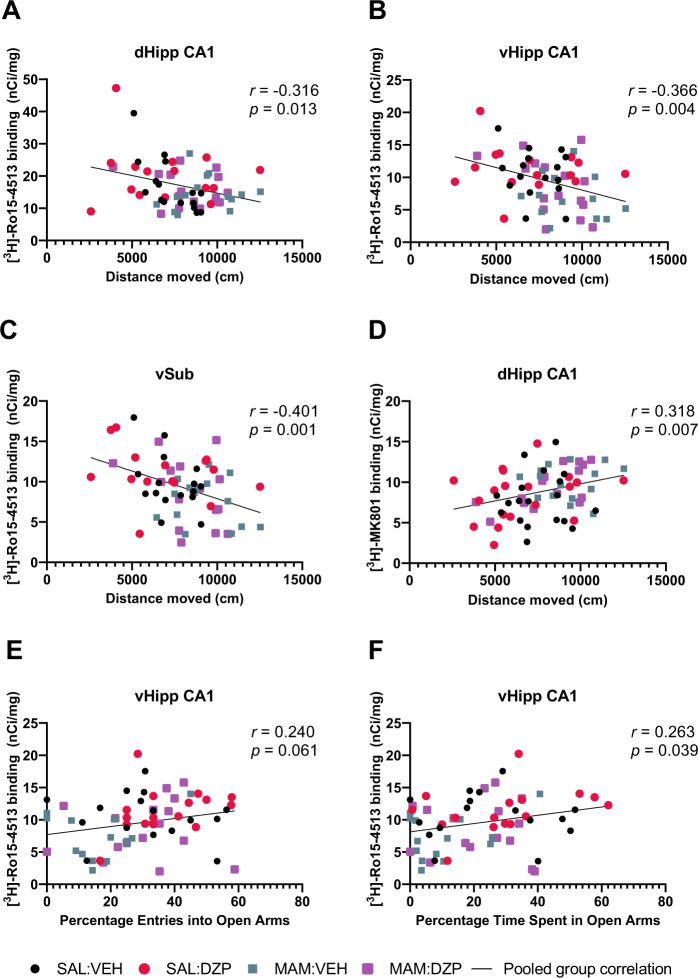
Significant autoradiography × behavior correlations for pooled groups. **A**–**C** [^3^H]-Ro15-4513 binding was inversely correlated with amphetamine-induced hyperlocomotion. **D** [^3^H]-MK801 binding was positively correlated with amphetamine-induced hyperlocomotion. **E**, **F** [^3^H]-Ro15-4513 binding was positively correlated with time spent in open arms, but correlation failed to reach significance with entries into open arms of the EPM.

Correlation between behavioral measures (AIH total movement, EPM time spent in open arms, EPM entries into open arms) and those receptor density measures that showed a significant group effect ([^3^H]-Ro15-3415 dHipp CA1, vHipp CA1, and vSub; [^3^H]-MK801 dHipp CA1) revealed that all correlations with AIH total movement were significant (Fig. 5). Lower [^3^H]-Ro15-4513 receptor binding was associated with greater locomotor response to amphetamine (dHipp CA1: *r* = −0.316, *p* = 0.013, *q* = 0.013, Fig. 5A; vHipp CA1: *r* = −0.366, *p* = 0.004, *q* = 0.006, Fig. 5B; vSub: *r* = −0.401, *p* = 0.001, *q* = 0.003, Fig. 5C). Meanwhile, higher [^3^H]-MK801 receptor binding in the dHipp CA1 was associated with higher locomotor response to amphetamine (*r* = 0.318, *p* = 0.007, Fig. 5D). In terms of EPM measures, vHipp CA1 [^3^H]-Ro15-4513 binding was significantly positively associated (*r* = 0.263, *p* = 0.039, Fig. 5F) with time spent in open arms, but a positive association with entries into open arms missed significance (*r* = 0.240, *p* = 0.061, Fig. 5E). No other significant correlations with EPM measures were found. Correlations were also run separately per group and are presented in the Supplementary Results (Table S3).

## Discussion

In this study, we sought to characterize GABAergic and glutamatergic receptor abnormalities in hippocampus using a well-validated neurodevelopmental model relevant to schizophrenia, and their potential modulation by peripubertal diazepam treatment. Our main finding was that MAM-treated rats displayed lower α5GABA_A_R density in key hippocampal sub-regions, indexed by lower [^3^H]-Ro15-4513 binding. This reduction appears to be specific to α5GABA_A_R, based on [^3^H]-Ro15-4513’s higher affinity to this receptor, and the lack of significant effects in nonspecific GABA_A_-BZR density as measured by [^3^H]-flumazenil. Furthermore, MAM treatment was associated with greater NMDAR density, specific to the dorsal hippocampus CA1 subfield. Importantly, these receptor abnormalities were linked to schizophrenia-relevant behavioral phenotypes, as both the α5GABA_A_R decrease and the NMDAR increase were associated with AIH. While we had predicted that peripubertal diazepam treatment would prevent the emergence of schizophrenia-relevant behaviors and normalize receptor density abnormalities identified by autoradiography, we observed only a partial behavioral rescue (i.e., anxiety-like behavior was rescued, but AIH was not) of the phenotypes and no treatment effects of diazepam on receptor densities.

Lower α5GABA_A_R density in the MAM model is consistent with our hypothesis, based on previous literature suggesting a key role for this subunit in correcting several aspects of the MAM-related phenotype, including dopamine-system hyperactivity [34, 41]. α5GABA_A_R binding in the hippocampus has been described in unmedicated schizophrenia patients by in vivo PET imaging using the same ligand [^11^C]-Ro15-4513 [28]. Our findings thus provide first evidence that a specific deficit in this receptor subtype is involved in MAM pathophysiology relevant to schizophrenia, using a translational imaging measure, and building on previous evidence that selectively targeting this subunit could potentially compensate for schizophrenia pathology [34]. In terms of mechanisms, lower α5GABA_A_R function in vHipp CA1 and vSub may underlie hippocampal hyperactivity in schizophrenia through disinhibition of glutamatergic pyramidal cell activity [34]. Neuroimaging studies in patients with schizophrenia and high-risk individuals have documented elevated cerebral blood volume/flow specifically in the CA1 subfield [13, 57, 58]. Moreover, elevated cerebral blood volume correlated with positive symptom severity [13, 58], consistent with the inverse relationship we observed between hippocampal α5GABA_A_R density and locomotor response to amphetamine, as α5GABA_A_R regulate tonic inhibition. Our findings also align with current predictive processing (i.e., active inference) accounts of schizophrenia. Briefly, false inference resulting in positive symptoms of schizophrenia is thought to result from an imbalance in the precision of prediction errors used for (Bayesian) belief updating [59–64]. Precision can be read as the predictability of prediction errors, which is thought to be encoded by the excitability or disinhibition of neurons encoding prediction errors (e.g., superficial pyramidal cells) on a synaptic level. Current evidence suggests that the positive symptoms of schizophrenia may represent a compensation for a failure to attenuate the precision of sensory prediction errors; i.e., a failure of sensory attenuation [65–70]. To compensate for the attenuation of sensory prediction errors, high-level prediction errors that update prior beliefs, such as those encoded in the hippocampus, may be augmented through disinhibition of pyramidal cells generating top-down predictions. Inhibitory interneurons play a particularly important role in this respect [71–73], which fits very closely with our findings.

Interestingly, anxiety-like behavior was exclusively associated with α5GABA_A_R in the vHipp CA1, as no associations were found with any of the other sub-regions or receptor types. Human and animal studies have suggested that hippocampal dysfunction originates in CA1 and spreads to the subiculum, from which the hippocampus then dysregulates ventral tegmental area dopamine neurons [9, 49, 74]. These findings may suggest that the ventral CA1 may be most intimately linked to psychosis pathophysiology and to the increased stress responsivity thought to be a risk factor for transition [75].

Within the dHipp CA1, we observed lower α5GABA_A_R and increased NMDAR. Implications of lower α5GABA_A_R in the dHipp CA1 remain unclear. Previous research by our group identified a dose-dependent change in α5GABA_A_R density in this region in response to chronic haloperidol exposure [29]. Hence, a possible explanation may relate to a disease-driven receptor abnormality in this region, which may explain the receptor increases in response to haloperidol [29] and provide another mechanism of action for antipsychotics. In terms of NMDAR, the observed increase in dHipp CA1 aligns with a prior autoradiographic study of human postmortem brain tissue from patients with schizophrenia [48]. Specifically, an overall increase of NMDAR in multiple regions including the hippocampus was found; however, only receptors in the putamen reached statistical significance. Kornhuber et al. [48] speculated that this increase may be partially due to effects of antipsychotic exposure; however, our findings suggest that this may not be the case. Due to the putative hypofunction of this receptor in the pathophysiology of schizophrenia [19], increased expression may be a compensatory mechanism that develops alongside schizophrenia progression. The negative correlation of α5GABA_A_R and the positive correlation of NMDAR with AIH would suggest that these receptors are moderately affiliated with behavioral abnormalities relevant to schizophrenia. However, unlike α5GABA_A_R in vHipp CA1, dHipp CA1 NMDAR were not related to stress measures (EPM). Given that the hippocampus is a functionally segregated structure, with vHipp (anterior in primates) playing a role in emotion and stress while the dHipp (posterior in primates) is involved with information processing [76], future studies including behavioral tasks dependent on dHipp, such as spatial memory and context discrimination [76, 77], will expand on our findings.

In terms of [^3^H]-flumazenil, we found no effects in GABA_A_-BZR density in MAM-treated animals. [^3^H]-flumazenil was used as a positive control to test the specificity of [^3^H]-Ro15-4513 to α5GABA_A_R. In combination with only partial evidence supporting a diazepam effect in the behavioral measures (i.e., only in EPM measures, not in AIH), these observations may reflect that repeated diazepam treatment failed to fully recover the model in our study, in contrast with previous work [35, 38]. In humans, an elevated anxiety response to stress is present in high-risk individuals and associated with the subsequent transition to psychosis [75]. Peripubertal diazepam in the MAM model is thought to prevent schizophrenia-like behavior by attenuating this heightened stress response [35, 38]. However, in humans, on occasion an uncommon (in <1% of patients) paradoxical reaction to benzodiazepines occurs: rather than displaying signs of sedation they become agitated, excited, and engage in emotional release and excessive movement [78]. Albeit rare, these reactions have been putatively linked to psychiatric disorders such as bipolar disorder and schizophrenia [78], but the mechanisms still remain elusive. Such reactions illustrate that diazepam acts on a functionally diverse system of feedforward and feedback connections, and synaptic and extrasynaptic receptors [20], and thus the effects of diazepam may not always be predictable.

There are some limitations to our study that should be noted. Firstly, we used AIH as an assay to index schizophrenia-like behavior; however, the analogousness to associative striatum hyperdopaminergia in humans has been debated [79–81]. AIH is rather thought to reflect ventromedial limbic striatal dopamine action and thus cannot be taken as a direct analogue of positive symptoms in animal models. Nonetheless, this behavioral assay is commonly used to study the efficacy of antipsychotic drugs [80]. Future studies using behavioral tests assessing salience attribution and selective attention, which are more directly related to associative striatum dopamine release [81], are warranted. Secondly, autoradiography was only performed at adulthood, as imaging of autoradiographs required termination of the animal and as such cannot be performed longitudinally in the same animals. Our experimental design allowed measuring behavior, drug effects and receptor densities in the same animals. However, this design does not allow tracking neurodevelopmental changes in α5GABA_A_R and NMDAR densities and their potential role MAM pathophysiology. Further in vivo studies using these tracers in the context of PET imaging will enable mapping of the trajectory of receptor abnormalities identified in our study.

In summary, the present findings implicate α5GABA_A_R abnormalities in schizophrenia-relevant pathophysiology, and provide new empirical support to the notion that the development of pharmacological agents with selectivity for hippocampal α5GABA_A_R may be a promising new therapeutic target to prevent schizophrenia-related deficits. As our report is, to our knowledge, the first to image GABA_A_R and NMDAR in the MAM model with comparable measures that can be used in humans, future translational studies imaging these receptor subunits in early psychosis are warranted to inform whether clinical interventions targeting this pathway may have the potential to prevent or delay the development of psychosis in vulnerable individuals.

## Supplementary information

Supplemental Material
